# Size of the Carotid Body in Patients with Cardiovascular and Respiratory Diseases Measured by Computed Tomography Angiography: A Case-Control Study

**DOI:** 10.1155/2021/9499420

**Published:** 2021-10-15

**Authors:** Sándor Csizmadia, Gergely H. Fodor, András Palkó, Erika Vörös

**Affiliations:** ^1^Affidea Hungary Ltd. Budapest, 44-46 Bókay János Street, Budapest H-1083, Hungary; ^2^Department of Medical Physics and Informatics, University of Szeged, Faculty of General Medicine, 9 Korányi Alley, Szeged H-6725, Hungary; ^3^Department of Radiology, University of Szeged, Faculty of General Medicine, 6 Semmelweis Street, Szeged H-6725, Hungary

## Abstract

**Objectives:**

Carotid bodies (CBs) play an important role in regulating sympathetic nervous system activity. Thus, they are likely to be enlarged in patients with certain cardiovascular and respiratory diseases. The aim of this case-control study was to verify this hypothesis using computed tomography angiography (CTA).

**Methods:**

We retrospectively analysed 141 CTAs including 16 controls, 96 patients with only hypertension (HT), 12 with HT and previous acute myocardial infarction (AMI), 9 with HT and heart failure (HF), and 8 with HT and chronic obstructive pulmonary disease (COPD). We assessed the data using analysis of variance, with *p* < 0.05 indicating significance.

**Results:**

CB average areas in the controls were 2.31 mm^2^ (right side (RS)) vs. 2.34 mm^2^ (left side (LS)). CB size was significantly enlarged in patients with HT: 3.07 mm^2^ (RS) (*p*=0.019) vs. 2.91 mm^2^ (LS) (*p*=0.002). If AMI (RS: 3.5 mm^2^; LS: 3.44 mm^2^) or HF (RS: 4.01 mm^2^; LS: 4.55 mm^2^) was associated with HT, the CB size was even more enlarged. COPD did not affect CB size (RS: 2.40 mm^2^; LS: 2.29 mm^2^).

**Conclusions:**

Our data showed that certain diseases with increased activity of the sympathetic nervous system were associated with significantly enlarged CBs.

## 1. Introduction

Carotid bodies (CBs) are small, paired organs that contain peripheral chemoreceptors and chromaffin cells. Richly supplied by blood vessels, CBs are located above the bifurcation of the common carotid artery. The amount of blood that flows through CBs is 20 times their weight per minute. Their main role is to sense the pressure of certain gases in the blood that passes through them, especially the partial pressure of O_2_ and CO_2_, as well as to monitor the pH level [1–4].

According to the literature, mitochondria in the glomus cells of CBs are sensitive to hypoxia. Therefore, if the arterial partial O_2_ pressure decreases, the central nervous system is informed, resulting in the ventilation being stimulated. In addition to chemoreceptors, baroreceptors and thermoreceptors are also present, and some studies suggest that they play a role in glucose homeostasis as well. CBs are essential to regulating respiration and the blood circulation [5–8]. With their chromaffin cells, CBs take part in relevant processes of the sympathetic nervous system. By producing adrenaline and noradrenaline, they compensate the hypoxaemia-induced direct vasodilatation effect, promoting better adaptation [9, 10].

The hyperactivity of the chromaffin cells and their increased mass have been observed in patients with certain cardiovascular and respiratory diseases. Furthermore, the pathological processes of the liver and some metabolic or haematological states have an influence on the size of CBs' cells. Several articles have described the enlargement of CBs but did not compare the extent of the enlargement in the different diseases [11–21].

Previous studies with small groups of patients have shown that we can identify CBs reliably by computed tomography angiography (CTA) and can determine their exact size. We can identify CBs by searching for an avidly enhancing structure in its characteristic location, at the inferomedial aspect of the carotid bifurcation [22, 23].

The aim of this case-control study was to verify the enlargement of CBs with the help of CTA in a large number of patients with the following conditions: hypertension (HT); heart failure (HF) and HT; acute myocardial infarction (AMI) and HT; and chronic obstructive pulmonary disease (COPD) and HT. The second aim of the study was to determine the extent of the enlargement of CBs in the presence of the abovementioned conditions. Our purpose was to compare the measured data in order to better understand the operation of CBs and draw conclusions about their functions.

## 2. Materials and Methods

### 2.1. Study Group

The first phase of our study was to collect the records of neck carotid CTAs performed in the centre of Affidea Hungary Ltd., Szeged, during a one-year period. In total, 512 examinations were performed. Then, the conditions of the patients were investigated precisely with the help of the hospital informatics system Medsolution (International System House (ISH) Ltd., Budapest, Hungary). The International Statistical Classification of Diseases and Related Health Problems coding system provided significant help in this research. In all, 150 patients were found with sufficient information relevant to the study. Some diseases influence the size of CBs, including lesions of the liver (e.g., liver cirrhosis and hepatitis) and haematological or metabolic diseases (e.g., lactate acidosis and haemoglobinopathy). As only two patients suffered from liver cirrhosis, it was not enough for statistical analysis, so we excluded them from the study. Exclusionary haematological diseases or lactate acidosis could not be verified in any of the patients. In seven cases, we found more than two of the examined illnesses. Finally, 141 patients met the previously determined criteria and were classified according their conditions. There were 16 controls. HT alone was identified in 96 patients, HT associated with AMI in 12 patients, HF and HT in 9 patients, and COPD associated with HT in 8 patients.

All patients who suffered from HT had essential HT, and it was proven further by the criteria of blood pressure measurements. In all questionable cases, the patient underwent 24-hour ambulatory blood pressure monitoring (ABPM). In case the patient had an episode with typical ECG signs and the typical blood test, with elevated troponin level and creatine kinase level, AMI was proven in the anamnesis. In case of HF, the diagnosis was proven by echocardiography and the increased level of N-terminal pro-B-type natriuretic peptide (NT-proBNP) in the blood test. COPD was identified if spirometry proved the diagnosis by the decrease of forced expiratory volume during the first second (FEV1) and the decrease of the Tiffeneau-Pinelli index below 70%. Detailed information about the study groups is given in Table 1.

### 2.2. Measurement and Evaluation

The examinations were performed by using a 64-slice GE LightSpeed VCT XTe CT Scanner (General Electric, Fairfield, Connecticut, USA). The slices were obtained in the helical mode with 1.25 mm slice thickness. Patients were scanned from the aortic arch to the frontal sinus. The contrast agent (Omnipaque 350-General Electric, Fairfield, Connecticut, USA) was injected intravenously via an 18-gauge peripheral venous catheter. We used an antecubital vein if possible. When it was not feasible, the contrast material was injected into the dorsal venous network of the hand, with 50 ml of it used for each examination. We first administered 15 ml of contrast material at a rate of 2.5 ml/s and then 35 ml at a rate of 3.0 ml/s, followed by a 25 ml saline flush.

We managed the process with the SmartPrep technique. We began the examination when the contrast agent appeared in the region of interest which was in the aortic arch. The scans were uploaded to the Picture Archiving and Communication System database of GE. One of the authors, who was unaware of the clinical conditions of the patients, executed the identification and measurement of CBs twice at different times.

**Figure 1:**
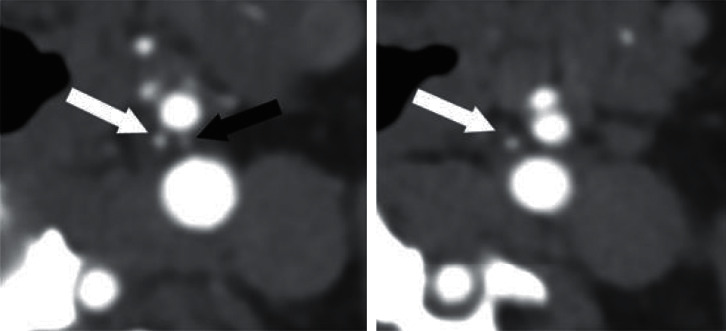
Images from a 53-year-old man in the control group (right: caudal image in which the small, avidly enhancing left carotid body (CB) (black arrow) is seen between the external carotid artery (smaller one on the ventral side) and the internal carotid artery (larger one on the dorsal side); left: a somewhat more cranial image in which the narrow branch of the external carotid artery (white arrows) is easily differentiated from the carotid body).

**Figure 2:**
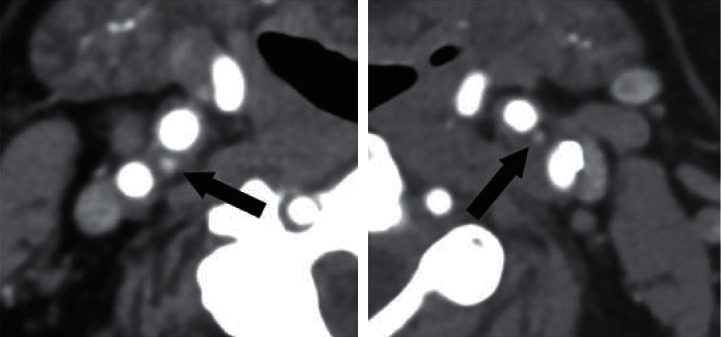
CTA shows moderately enlarged carotid bodies (arrow) on both sides in a 54-year-old woman who suffers from high blood pressure.

**Figure 3:**
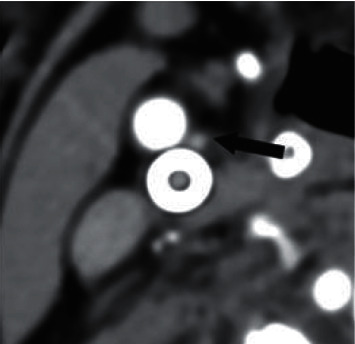
CTA axial slice from an 84-year-old man who had experienced a previous AMI and suffers from HT. The patient also had had a carotid stent implanted, but the enlarged CB (arrow) is still easily identified.

An axial scan was chosen for evaluation in each case because CBs could be best identified on axial scans. We used magnification if necessary. We measured the anteroposterior and the latero-lateral diameters of CBs on both sides on two occasions. The two measured areas were then averaged and used as final data.  Figure 1 shows the identification processes. Figures 2 and 3 show examples from the examined groups.

### 2.3. Statistical Analysis

The analysis and evaluation of data were performed with SigmaPlot software package (version 13; SYSTAT Software Company Inc., San Jose, CA, USA). The Shapiro−Wilk test was used to test data for normality. The Kruskal−Wallis one-way analysis of variance on ranks test was executed to compare the results of all of the groups with each other, with *p* < 0.05 indicating significance. Dunn's multiple comparison procedure was applied to compare the groups. We also compared the measured data of the two measurement series (intraobserver variability) with the help of the Wilcoxon test.

## 3. Results

Successful identification of CBs was the primary criteria in the evaluation of the measured data. The identification was considered successful only if CBs were found on two separate occasions. The measured data were recorded and evaluated rigorously on both the right side (RS) and the left side (LS). On the RS, CBs were precisely detected in 129 of the 141 patients, with a success rate of 91%. Identification was even more successful on the LS, where they were clearly measured in 134 patients, with a 95% success rate.

We examined the two measurement series and compared them with each other to determine intraobserver variability. The average size ± standard deviation of the CB on the RS was 3.05 ± 0.97 mm^2^ for the first measurement series and 3.04 ± 0.89 mm^2^ for the second one. On the LS, the average CB size was 2.94 ± 0.88 mm^2^ for the first measurement series and 2.95 ± 0.87 mm^2^ for the second series. No significant intraobserver variability was found for the cross-sectional areas (*p*=0.54 for the RS and *p*=0.42 for the LS).

In the control group, the average size of the CB was 2.31 ± 0.82 mm^2^ on the RS and 2.34 ± 0.66 mm^2^ on the LS. There was no significant difference between the two sides. The CB was larger in the HT-only patients (3.07 ± 0.81 mm^2^ on the RS and 2.91 ± 0.74 mm^2^ on the LS) than in the control group. On the RS, there was a significant difference in its size between the HT-only patients and the control group (*p*=0.019). On the LS, the difference was clear, but it was not quite at the level of significance (*p*=0.079). In patients with AMI and HT, the average size of the CB was 3.5 ± 0.74 mm^2^ on the RS and 3.44 ± 0.72 mm^2^ on the LS. The measured data showed exactly the same tendency as in our previous assumptions: the CB was larger in patients with a prior heart attack and HT than in the HT-only patients or the controls. In the latter case, the difference was significant on both sides (*p*=0.002 in both cases). Compared with patients with HT, the difference is detectable but not significant (*p*=0.583 on the RS and *p*=0.180 on the LS). Thus, patients with HF and HT had the largest CBs, with an average dimension of 4.01 ± 1.39 mm^2^ on the RS and 4.55 ± 1.24 mm^2^ on the LS. Compared with the control group, significance was obvious on both sides (*p*=0.003 on the RS and *p* < 0.001 on the LS).

The average size of the CB in patients with COPD and HT was 2.40 ± 0.54 mm^2^ on the RS. It can be seen that the CB is smaller in these patients than in those who had HT alone. The size is almost the same as in the control group. No significant difference could be observed among the three groups. Statistically, the CB in patients who had COPD and HT was significantly smaller than that in patients with HT and HF and in those with HT and AMI (*p*=0.026 in the first case and *p*=0.033 in the second). Measurements on the LS showed the same results: the size of the CB was 2.29 ± 0.27 mm^2^. We found a significant difference with the HT and HF group (*p* < 0.001) and with the HT and AMI group (*p*=0.005). We also compared the COPD and HT group with the control patients and the HT-only group on the LS. We could not identify a significant difference in either case.

**Figure 4:**
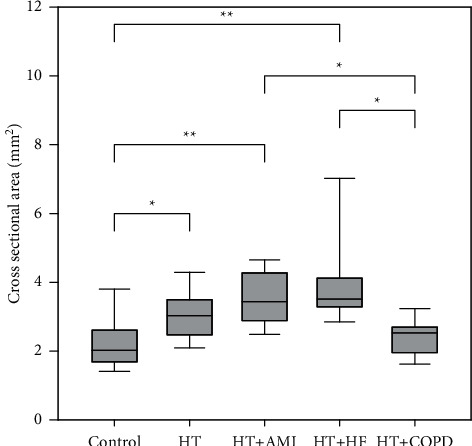
Our results and significant pairs ( ^*∗*^*p* < 0.05 and  ^*∗∗*^*p* < 0.01) for the right-side carotid bodies with the standard deviations. HT, hypertension; AMI, acute myocardial infarction; HF, heart failure; COPD, chronic obstructive pulmonary disease.

**Figure 5:**
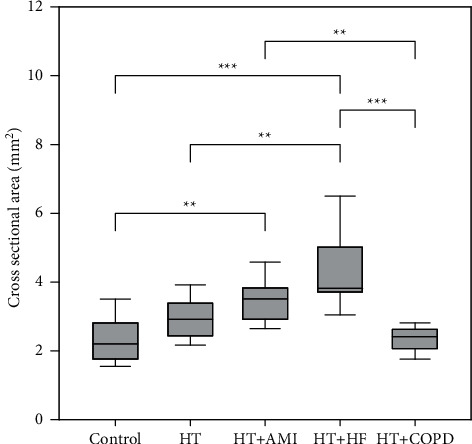
Significant differences ( ^*∗*^*p* < 0.05,  ^*∗∗*^*p* < 0.01, and  ^*∗∗∗*^*p* < 0.001) and the measured cross-sectional sizes of the left-side carotid bodies.

The right and left sides were compared with each other in every examined group, with no significant differences found. The number of identified CBs, the success rate of identification, and the average size of CBs for the right side are shown in Table 2, and those for the left side are shown in Table 3. Figures 4 and 5 show the results and the significant pairs. The significant pairs and *p* values for every case are shown in Table 4.

## 4. Discussion

It is well known that CBs play an important role in regulating the respiratory and blood circulation systems. They are the most important chemosensors, baroreceptors, and thermoreceptors in the human body besides the brain. These sensors instigate the flow of information to the central nervous system about possible hypoxia. Their chromaffin cells participate in the activity of the sympathetic nervous system as well. As it is known, these cells are mostly located in the medulla of the adrenal gland. They produce noradrenaline and adrenaline, thereby playing a role in stimulating the sympathetic nervous system. It is important to compensate for the hypoxaemia-induced direct vasodilatation effect, thereby helping improve adaptation [3–10].

CBs enlarge significantly in the presence of cardiovascular diseases such as HT or HF. Moreover, it seems that respiratory diseases such as COPD and pathological processes of the liver as well as some metabolic or haematological states also influence the size and histologic structure of CBs. In these cases, the sympathetic nervous system might be overactivated, so the number of the chromaffin cells in this system could be increased. The role of CBs in the formation, progression, and maintenance of these diseases is still not fully clarified. The main problem is that some of the available data about CBs are derived from postmortem examinations, animal experiments, and studies with small groups of patients [7, 9, 12–14].

In patients with HT or HF or following AMI, the sympathetic tone could be intensified. In this study, we intended to evaluate the morphological signs related to this role of the CB based on alteration of its size. Hence, the carotid CTA examinations of 141 patients were reanalysed retrospectively. It was clearly proved that CBs could be reliably identified using this technique, with a success rate of >90%. Advanced calcification around the bifurcation or on a previously built-in metal stent makes measurement more difficult, as either situation deforms the normal anatomic conditions. As can be seen in Figure 3, there were some cases in which the size of the CB could be clearly measured despite the complicating conditions. Jie et al. [12] examined CBs by ultrasound. Even though they had studied a larger group, we could achieve a higher success rate of identification by our CTA study.

Our results confirmed that CBs are significantly enlarged in patients with HT. Previously, Habeck et al. [13] and Honig et al. [14] verified the enlargement of CBs in rats with high blood pressure. Presumably, because of the permanently increased activity of the sympathetic nervous system, the volume and number of chromaffin cells increase, thereby increasing the size of the CB. The significant enlargement proven by the measurements verified our assumptions and confirmed the results of previous studies. Sreejit et al. [23] proved that the volume of CBs also grows in patients with both HT and HF. Based on previous pathologic and pathophysiologic data, it is obvious that the simultaneous presence of the two diseases would further increase the sympathetic activity. Our CTA results verified this tendency of increase in vital situations. We measured significantly larger volumes on both sides in patients with both HT and HF versus the control group and the HT-only group. We also found that if the patients had not only HT but also a previous AMI, CBs had enlarged, although its size did not reach that in the HT and HF group. Perhaps the durations of the two diseases were responsible for the difference in the amount of enlargement.

Based on previous studies, we assumed that the diameter of CBs enlarges in patients with COPD. Our measured data, however, did not corroborate that tendency. The recorded sizes on both sides were nearly the same as those in the control group with no significant difference. In a recent article, Vinhaes et al. [24] proved histologically that the construction of cells and their proportions change not only in COPD patients but also in those with acute respiratory distress syndrome. We thus assume that the increase in size is not the main determining change in COPD. Histologic remodelling is probably much more important in these diseases. Another reason we may not find larger areas is that COPD is an umbrella designation under which various illnesses reside. These different types of disease could affect CBs in different ways and to different degrees. Further analysis of the relations could be useful.

There are some limitations of our study, which we intend to solve in the future. First, it was not possible to measure catecholamine production by CBs directly, so we judged this function according to CB size. Second, although our study is based on a large group of patients, some of the subgroups were small. Thus, we have the data about medication of patients and the stage or grade of examined diseases; these could not submit accurate statistical analysis. It would be interesting to examine the nontreated or the not appropriately treated cases in separate groups. This evaluation could provide evidence to prove that the size of CBs correlate with the medical treatment and with the severity of the disease. It could be the subject of our following study.

The aims for the future are to widen the group of examined patients and to execute prospective measurements. These additions would provide more accurate information from the patients, and the abovementioned obstacles could be eliminated. Further improvement could be achieved if the resolution was enhanced and if the measurements were done on, for example, MRI scans as well or were compared with them [25–27].

## 5. Conclusion

We proved, using CTA, that CBs are indeed enlarged in patients with diseases that cause the sympathetic nervous system's operation to be intensified. Based on our measurements in patients with HT, the diameter of CBs is significantly larger than that of CBs in the average, healthy individuals. Its size further enlarges in patients who in addition to HT also have HF or AMI in the anamnesis. Taking our measurements into consideration allows the possibility of drawing conclusions about the physiologic and general state of the patient after analysing his or her anatomic structures on CTA. This protocol could provide us and clinicians additional information about the patient. In the future, it could be interesting to increase the number of patients and to analyse precisely the treatment used. It would allow the possibility of determining the efficacy of treatment of these diseases.

## Figures and Tables

**Table 1:** Data for all patient groups.

Patient groups	Control	HT	AMI + HT	HF + HT	COPD + HT	Altogether
Number of patients	16	96	12	9	8	141
Female	7	47	3	5	4	66
Male	9	49	9	4	4	75
Average age (Min–Max in years)	48.63 (21–78)	65.79 (29–88)	63.58 (44–85)	65.78 (29–88)	65.38 (58–72)	61.83 (21–88)

HT, hypertension only; AMI + HT, previous acute myocardial infarction and hypertension; HF + HT, heart failure and hypertension; COPD + HT, chronic obstructive pulmonary disease and hypertension.

**Table 2:** Success rate of identification and comparison of cross-sectional areas of the right CB.

Right side	Control	HT	AMI + HT	HF + HT	COPD + HT
Identified CBs	15	89	11	7	7
Success rate of identification (%)	93.75	92.7	91.67	77.78	87.5
Mean size of the CB (mm^2^)	2.31	3.07	3.56	4.01	2.40
Standard deviation (±)	0.82	0.81	0.74	1.39	0.54

**Table 3:** Success rate of identification and comparison of cross-sectional areas of the left CB.

Left side	Control	HT	AMI + HT	HF + HT	COPD + HT
Identified CBs	16	91	12	7	8
Success rate of identification (%)	100	92.71	100	77.78	100
Mean size of the CB (mm^2^)	2.34	2.91	3.44	4.55	2.29
Standard deviation (±)	0.66	0.74	0.72	1.24	0.27

**Table 4:** Significant pairs on the right and left sides using the *p* value.

Right side		*p*	Left side		*p*
Control	HT	0.019	Control	AMI + HT	0.002
Control	AMI + HT	0.002	Control	HF + HT	<0.001
Control	HF + HT	0.003	HT	HF + HT	0.005
COPD + HT	AMI + HT	0.033	COPD + HT	AMI + HT	0.005
COPD + HT	HF + HT	0.026	COPD + HT	HF + HT	<0.001

## Data Availability

Data are available on request.

## Abbreviations

ABPM: Ambulatory blood pressure monitoring
AMI: Acute myocardial infarction
CB: Carotid body
COPD: Chronic obstructive pulmonary disease
CTA: Computed tomography angiography
FEV1: Forced expiratory volume during the first second
HF: Heart failure
HT: Hypertension
LS: Left side
RS: Right side
NT-proBNP: N-terminal pro-B-type natriuretic peptide.

## Additional Points

*Highlights.*(1) The carotid body plays a role in sympathetic nervous system activity;(2) the size of the carotid body can be reliably measured by computed tomography angiography; and (3) the carotid body is enlarged in certain diseases with increased sympathetic activity.
